# Educating the clinical trainer: professional gain for the trainee? A controlled intervention study in general practice

**DOI:** 10.1007/s40037-014-0142-z

**Published:** 2014-10-23

**Authors:** H. G. A. Ria Jochemsen-van der Leeuw, Nynke van Dijk, Wilfried de Jong, Margreet Wieringa-de Waard

**Affiliations:** 1 Department of General Practice/Family Medicine, Academic Medical Center, University of Amsterdam, PO Box 22700, 1100 DE Amsterdam, the Netherlands; 2 Department of General Practice/Family Medicine, Academic Medical Center, University of Nijmegen, Nijmegen, the Netherlands

**Keywords:** Faculty development, Medical education—educational assessment, Role modeling, Obesity, Primary care

## Abstract

The aim of this study was to establish whether a ‘teach-the-trainer’ course leads to improvements in, firstly, the knowledge and attitude of clinical trainers and their trainees, and, secondly, the role model behaviour of the clinical trainers. A controlled intervention study was performed with GP trainers and GP trainees from four training institutes in the Netherlands. Clinical trainers in the two intervention institutes received two 3-h training sessions on weight management, focusing on knowledge and attitudes towards obesity, and on conveying the correct professional competency as a positive role model for trainees. This was measured using questionnaires on knowledge, attitude, and role model behaviour (the role model apperception tool; RoMAT). GP trainers showed an increase in knowledge and several characteristics could be identified as being related to positive role model behaviour. A small correlation was found between the trainer’s score on the RoMAT and the attitude of the trainee. A teach-the-trainer course in which knowledge, attitudes, and role modelling are integrated proved to be a first step toward improving the knowledge of clinical trainers, but did not result in a measurably better professional outcome for the trainee, maybe due to a more objective level of assessment.

## Background

Clinical trainers in general practice (GP) participate in continuing professional development (CPD) and faculty development (FD) courses at training institutes for GP speciality training. These educational interventions are expected to improve the quality of trainee-education in clinical practice by way of a cascade effect, representing the transfer of information from course to clinical trainer (Step 1) to training practice (Step 2) and to trainee (Step 3).

**Fig. 1:**
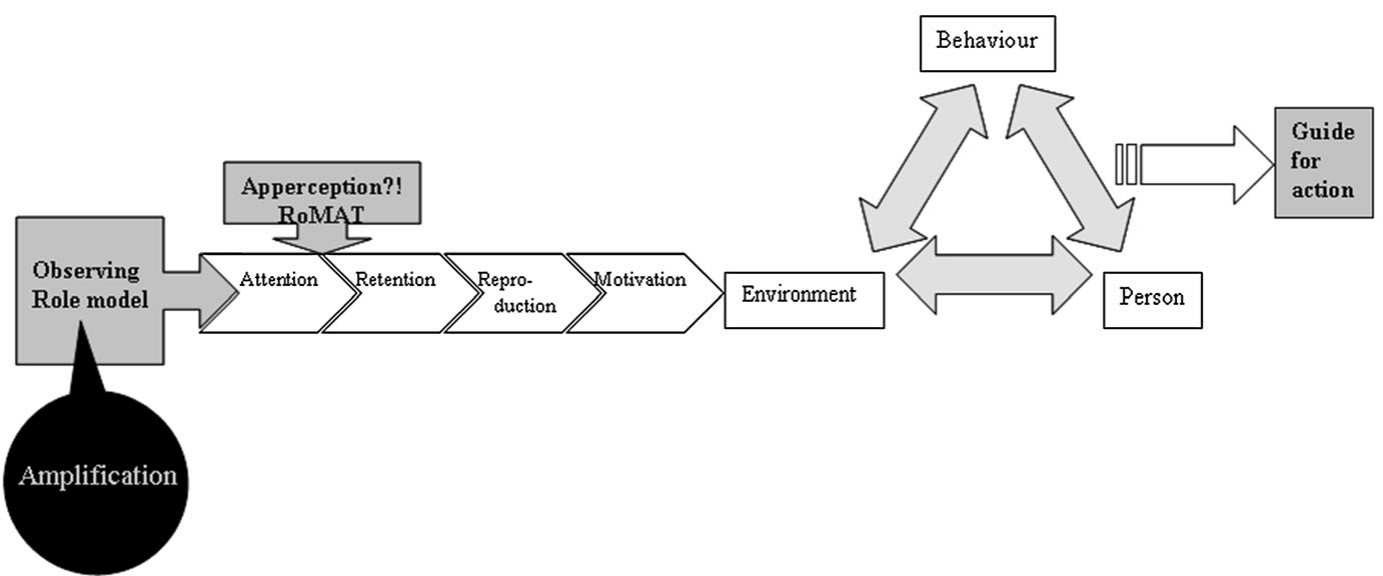
Model based on the social learning theory according to Bandura, supplemented by the steps of apperception (by using the RoMAT) for the trainee and amplification of the role model behaviour for the trainer

This is important because, during their clinical training [1], trainees are expected to grow into their future role as independent and competent professional physicians capable of providing high-quality patient care [2]. They learn clinical reasoning skills and behaviour not only at special teaching moments where the clinical trainer functions as a teacher and mentor, but also during everyday practice when they observe their clinical trainers as role models in the clinical workplace [3–6]. Stegeman emphasizes that role modelling is a major means of clinical workplace learning. [7] Bandura, in his social learning theory, explains learning by observation through modelling (Fig. 1) [8]. By adding the step of ‘apperception’ (the process whereby the perceived qualities of the trainer are related to past experiences) to this process, trainees can assess the role model behaviour of their trainers more consciously (Fig. 1) [9].

In order to ‘amplify’ their role model behaviour, clinical trainers need to develop role modelling awareness [10] and to be explicit about what they are modeling. [7] To prepare clinical trainers for their roles as teachers and role models, they receive structural training in both medical content and didactic topics. It is unknown, however, whether both these elements are conveyed by the trainers to their trainees, and which characteristics of the trainer influence this process [11]. It is known that ‘teach-the-teacher’ courses focusing on didactic skills result in better teaching/coaching behaviour, and are therefore beneficial for trainees [12–14]. Consequently we may expect that training for clinical trainers [15–19] that focuses on knowledge and attitudes regarding a medical subject, with training in conveying knowledge and an appropriate professional attitude to the trainee as a positive role model [6], may also improve the professionalism of the trainer and how this is conveyed to the trainee.

The research questions of this study were therefore the following:

- Does a teach-the-trainer course addressing the knowledge, attitude and the role model function of the trainer lead to an improvement in the knowledge and attitude of both the trainer (Step 1 of the cascade) and the trainee (Step 3), and in the role model behaviour of the trainer (Step 2)?
- Which characteristics of the trainer/trainee are related to larger improvements in knowledge, attitude, or role modelling scores? (Step 1 and 2)
- Does positive role model behaviour by the trainer affect the performance of the trainees? (Step 3)

## Methods

This study was performed as a controlled intervention study, with a training course serving as the intervention.

### Context

GP trainers in the Netherlands spend 4 days a week supervising GP trainees in their own practices during the first and third years of the three-year GP training programme. In their second year, trainees work in a variety of clinical settings, supervised by clinical trainers. Trainees spend one day a week, and trainers 8 days a year at one of the eight institutes for GP speciality training, where they are taught by GPs and behavioural scientists (both to be referred hereafter as ‘teachers’). This ‘training for trainers’ consists of half-day or full-day courses that aim to improve the competences of the trainer, thereby assuming to improve the clinical training given to trainees.

### Participants

GP trainers and their trainees at four institutes for GP speciality training participated in this study. The intervention groups were located in Amsterdam (mandatory participation) and Nijmegen (voluntary participation), and the control groups in Leiden and Maastricht. The participants were approached between January and September 2012.

We included the control groups to correct for any changes in knowledge and attitudes attributable to publicly available information published during the study period.

All participants were informed about the nature of the study, that participation in the study (not training) was voluntary, and that questionnaires would be coded in order to prevent responses from being traceable to individual respondents. The trainers and trainees signed informed consent forms. These forms and other identifiers were stored in a locked cabinet by the head of the research department (MWdW). Ethical approval for this study was obtained from the Ethical Review Board of the NVMO (Dutch Association for Medical Education).

### Intervention

We organized a combined CPD/FD course introducing a new self-management method for the treatment of obesity.

The aim of the training course was, firstly, to acquire knowledge about obesity and its treatment, secondly, for participants to become aware of their own attitude toward overweight patients, and, thirdly, to emphasize the importance of conveying knowledge and the correct attitude to trainees by serving as positive role models. The training took place in groups of 15–20 trainers and consisted of 3 h of education at the start of the intervention and 3 h, 3 months later. The duration was the same as standard GP training courses in the Netherlands, taking into account the limited available time for CPD due to the demanding responsibilities of general practice in terms of patient care. However, several previous studies had shown that 6-h teaching skills courses significantly improved residents’ teacher ratings [20, 21].

The training course comprised interactive presentations, peer discussion about case histories and statements, self-reflection regarding attitudes and exercises aimed at practising how to convey professional competencies to trainees. All participants received the necessary materials, such as booklets for patients, to enhance implementation in daily practice. We used these mixed didactic and interactive training sessions because a previous review showed that these can improve professional practice and health care outcomes, provided the focus is on outcomes that are likely to be perceived as serious [22].

We chose the subject of weight management because of its importance in preventing health problems in patients [23, 24]. However, GPs are still unsure about implementing this policy. Trainees therefore experience serious difficulties in becoming competent in this subject since they are influenced by the negative role model behaviour of clinical trainers [25]. New guidelines for GPs in the Netherlands were published on this subject shortly before the intervention [26], and a new evidence-based [27] intervention was introduced for weight management in GP practice (the minimal intervention strategy for obesity [28]), addressing the barriers to implementing weight management [29] using booklets for patients. All this led to the assumption that an intervention focusing on weight management would fulfil the learning needs of trainers and trainees, and could lead to significant improvements in terms of knowledge and attitudes, and possibly bring about changes in behaviour in this area.

### Outcome measures

To evaluate the study we used questionnaires on:

- *Characteristics* We asked the participants to record the characteristics of their practices and themselves, especially their weight and weight-change in the previous year, as these influence motivation and self-efficacy in treating patients with obesity [30–32].
- *Knowledge* We designed a questionnaire consisting of 40 multiple-choice questions (the minimum for a reliable MCQ exam [33]) on weight management. We designed three equivalent questionnaires to ensure that each participant would complete an alternative list of questions every time. The questions were based on GPs’ guidelines on obesity [26, 34–36], and reviewed by two experts.
- *Attitudes* We translated an instrument measuring attitudes toward weight management, using forward–backward translation. This self-assessment instrument, consisting of 20 items scored on a 5-point Likert scale, with higher scores representing a more positive attitude, has not been formally validated, but has already been used successfully in previous studies [37, 38] (see Table 5 in Appendix 1).
- *Role modelling* We developed and validated a tool for assessing the role model behaviour of clinical trainers: the RoMAT [39] (see Table 6 in Appendix 2). This tool was developed on the basis of a systematic review of the literature [9] aimed at identifying the attributes of good role models. It consists of 17 items scored on a 5-point Likert scale, split into two components: ‘Caring Attitude’ and ‘Effectiveness.’ Both components include an equal number of items addressing personal, teaching, and clinical qualities, with high reliabilities (Cronbach’s alpha 0.92 and 0.84, respectively).
  To evaluate the extent to which the trainers were aware of their role model behaviour, we also asked the GP trainers to score themselves, and the trainees to score their trainers as role models using a 5-point Likert scale.
- *Behaviour* We asked trainers and trainees whether they discussed the subject of the intervention and whether they used the booklets in their practice as an objective measurement of implementation in daily practice.

### Assessment (Fig. 2)

The GP trainers at the intervention institutes completed questionnaires on knowledge and attitude three times: before the intervention (T1), immediately after the intervention (T2), and three months after the intervention (T3). The GP trainers at the control institutes completed these questionnaires only at T1 and T3, since no change was expected in the scores at T2 without an intervention. All the trainees completed two questionnaires assessing their own knowledge and attitude, as well as the role model behaviour of their clinical trainer, at T1 and T3. Trainers and trainees answered the questions on practice behaviour only at T3.

**Fig. 2:**
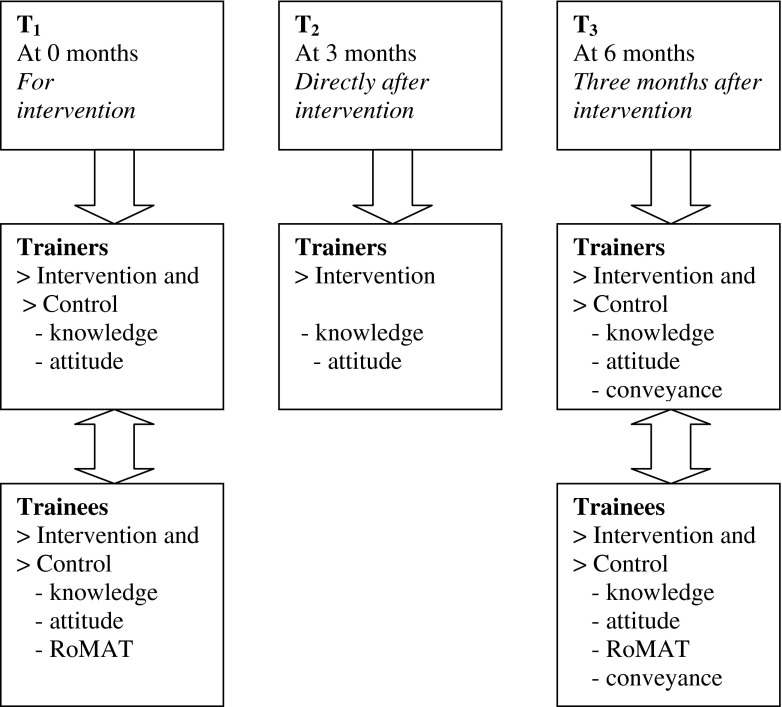
Schematic representation of the assessment

### Analyses

Data analysis was performed using SPSS version 20 (IBM SPSS Statistics, IBM Corp., Armonk, NY, USA). We used descriptive statistics to report the characteristics of the participants and training practices.

- We analyzed the change in scores in the intervention groups with a repeated-measures design, and compared the scores of the intervention and control groups using a one-way ANOVA. We calculated the effect sizes for all comparisons (ES = M_i_ − M_c_/SD_c_) [40].
- We calculated the correlation coefficient for the question about being a positive role model.
- Using one-way ANOVA, we analyzed the influence of the characteristics of the trainer on the increase in their scores and we did the same for the scores of the trainees.
- We investigated the relationship between the role model behaviour of the trainers and the individual test performances of all trainees in the intervention group (both the mandatory and voluntary ones) using Pearson’s correlation coefficient r at T3. Using univariate linear regression, we analyzed the influence of role model of the GP trainer on the T3 scores of the trainees.

Finally, to investigate the two items on behaviour, we used McNemar’s test for the question of discussing the subject and descriptive statistics for the question of distributing the booklets.

## Results

### Response

Only the GP trainers who attended the central curriculum days participated, and not every participant was present on all days: 184 (75 %) GP trainers participated in the mandatory group, 25 in the voluntary group, and 171 (67.1 %) trainers in the control group. The characteristics of the respondents and their practices varied in each group (Tables 1, 2).

**Table 1:**
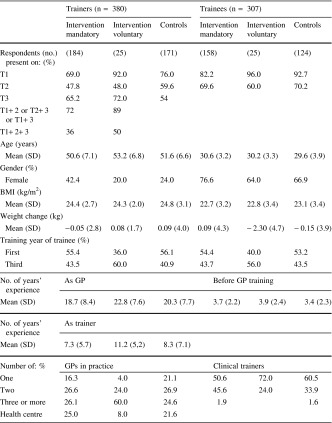
Personal characteristics of the respondents

**Table 2:** Characteristics of GP training practices

	Intervention mandatory	Intervention voluntary	Controls
Number of patients in practice (%)			
<2,000	17.9	32.0	9.4
2,000–2,500	29.3	44.0	34.5
>2,500	51.1	20.0	52.0
Location of practice (%)			
Village	17.9	32.0	32.2
Small town	9.2	4.0	12.3
City	29.3	28.0	29.8
Big City	42.4	28.0	22.8
Trainer/trainee couples (%)			
♂ + ♂	13.8	40.0	21.7
♀ + ♀	32.5	20.0	17.4
♀ + ♂/♂ + ♀	53.6	40.0	60.8

#### Improvements in knowledge, attitudes, and role model skills (Table 3)

In both intervention groups, knowledge scores increased significantly, and persisted to T3 (Step 1). Because of the small number of participants in the voluntary intervention group, their results were omitted from the comparative analyses. The knowledge scores of the trainees in the control group at T3 were significantly (p =  .02) higher than those of the intervention group, but the effect size (p = .37) was of little significance. [41] (Step 3)

**Table 3:** Mean scores on the questionnaires of trainers and trainees; score differences between T1 and T3 on knowledge, attitude and role modelling for each group; effect sizes for the differences between intervention and control groups

		Trainers			Trainees		
		Intervention mandatory	Controls	Intervention voluntary	Intervention mandatory	Controls	Intervention voluntary
Knowledge Mean (SD)	T1	16.5 (3.8)	15.9 (3.0)	16.2 (3.7)	15.1 (3.7)	15.5 (3.5)	14.9 (4.4)
	T2	18.2 (4.1)		22.4 (3.6)			
	T3	18.0 (4.3)	15.1 (3.6)	19.0 (3.7)	15.4 (3.9)	16.9 (4.1)	16.7 (2.6)
Mean Δ^a^	T3–T1	1.5	0.8	2.8	0.3	1.4	2.2
p Δ^a^ (Int-Cont)		0.00*			0.02*		
ES^b^		0.81			0.37		
Attitude Mean (SD)	T1	3.3 (0.2)	3.3 (0.2)	3.3 (0.3)	3.3 (0.3)	3.3 (0.2)	3.3 (0.2)
	T2	3.4 (0.2)		3.3 (0.2)			
	T3	3.4 (0.3)	3.3 (0.2)	3.7 (0.8)	3.3 (0.3)	3.3 (0.3)	3.5 (0.7)
Mean Δ^a^	T3–T1	0.1	0.0	0.4	0.0	0.0	0.2
p Δ^a^ (Int-Cont)		0.15			0.43		
ES^b^		0.50			0.00		
RoMAT Caring Attitude Mean (SD)	T1	4.3 (0.6)	4.3 (0.5)	4.4 (0.5)			
	T3	4.3 (0.5)	4.3 (0.5)	4.3 (0.6)			
Mean Δ^a^	T3–T1	0.0	0.0	−0.1			
p Δ^a^ (Int-Cont)		0.87					
ES^b^		0.00					
RoMAT Effectiveness Mean (SD)	T1	4.1 (0.5)	4.0 (0.6)	4.2 (0.5)			
	T3	4.1 (0.5)	4.1 (0.5)	4.0 (0.5)			
Mean Δ^a^	T3–T1	0.0	0.1	−0.2			
p Δ^a^ (Int-Cont)		0.48					
ES^b^		0.00					

*Int* intervention, *Cont* controls

^a^Change in score T3–T1

^b^Effect size = Mean_iintervention_ − Mean_control_ **/**SD_control_ (Hojat 2004) [40]

* Significant differences at p < 0.05

There was no correlation (r = −.010, p = .94) between the rating of the GP trainer and the rating of the trainee on the general question of whether the trainer was a positive role model. Conversely, the ratings of the trainees on the role model question correlated positively with the scores of the same trainees on the ‘Caring Attitude’ (r = .54, p < .001) and ‘Effectiveness’ (r = .63, p < .001) components (Step 2).

#### Influence of the respondents’ characteristics

Although no clear pattern emerged from the data, there were a few significant effects of characteristics on the change in scores (ΔT3–T1) of the GP trainers (Table 4). GP trainers who had recently lost weight, improved their knowledge more than those whose weight was stable or increasing [30, 31], showing that a topic with personal relevance motivates learning. Also, less experienced GP trainers improved their attitude more than experienced trainers, while more experienced trainers improved their role model behaviour in the ‘Effectiveness’ component. By contrast, GP trainers who worked alone in their practices showed better role model behaviour in the ‘Caring Attitude’ component than GP trainers who worked in a practice with others. GP trainers with fewer patients in their practices improved more on both the ‘Caring Attitude’ and the ‘Effectiveness’ components, compared with colleagues with over 2,500 patients. No significant influence was found of the trainees’ characteristics on their scores.

**Table 4:** Influence of the characteristics—personal, as a trainer and as a physician—of the GP trainers on the changes in their scores before and after the training, compared with Bonferroni’s procedure

Trainers (n = 80)	Knowledge Δ^a^		Attitude Δ^a^		RoMAT Δ^a^ Caring Attitude		RoMAT Δ^a^ Effectiveness	
	p mean	95 % CI	p mean	95 % CI	p mean	95 % CI	p mean	95 % CI
Weight change	0.03*	*Bf 1*<>*2*	0.46		0.32		0.94	
(1) <0	4.5	2.30–6.70	0.05	−0.07 to 0.16	0.23	−0.25 to 0.71	0.07	−0.21 to 0.36
(2) =0	1.3	−0.33 to 2.90	0.11	0.04–0.18	−0.01	−0.19 to 0.16	0.20	−0.15 to 0.19
(3) >0	1.5	−0.10 to 3.10	0.08	−0.06 to 0.13	−0.00	−0.13–0.13	0.02	−0.14 to 0.17
Years of experience as trainer	0.14		0.04*		0.25		0.05*	
(1) <6	3.0	1.50–4.40	0.12	0.05–0.20	−0.03	−0.25 to 0.18	−0.07	−0.24 to 0.10
(2) ≥6	1.5	0.09–2.90	0.01	−0.06 to 0.08	0.10	−0.02 to 0.22	0.13	0.01–0.25
No. of GPs in practice	0.68		0.95		0.02*	*Bf 1*<>*2*	0.08	
(1) 1	2.3	0.37–4.20	0.08	−0.02 to 0.18	0.37	0.02–0.71	0.17	−0.10 to 0.45
(2) 2	2.1	−0.00 to 4.10	0.06	−0.04 to 0.17	−0.16	−0.43 to 0.11	−0.18	−0.43 to 0.06
(3) ≥3	0.9	−0.95 to 2.60	0.05	−0.09 to 0.19	0.01	−0.10 to 0.25	0.08	−0.11 to 0.26
(4) Health centre	2.2	−0.04 to 4.50	0.03	−0.07 to 0.13	0.06	−0.06 to 0.18	0.12	−0.07 to 0.31
No. of patients in practice	0.81		0.30		0.04*	*Bf 2*<>*3*	0.01*	*Bf 1*<>*3*
(1) <2000	1.5	−1.10 to 4.00	−0.02	−0.14 to 0.10	0.14	−0.04 to 0.33	0.30	0.10–0.50
(2) 2,000–2,500	2.5	0.35–4.60	0.10	−0.00 to 0.20	0.22	−0.04 to 0.48	0.14	−0.07 to 0.35
(3) >2,500	2.1	0.77–3.50	0.06	−0.01 to 0.13	−0.10	−0.25 to 0.05	−0.10	−0.23 to 0.03

*Bf* Bonferroni

^a^Change in score T3–T1

* Significant differences at p < 0.05

#### Influence of role model behaviour on the trainees

There was a very small, but significant, correlation at T3 between the ‘Effectiveness’ score of the trainers and the attitude score of the trainees, r = .201, p = .027, indicating that GP trainers with better role model behaviour on the ‘Effectiveness’ scale were associated with GP trainees with the highest scores on attitude.

Regression analysis with role model behaviour as the independent variable and the attitude of the trainee as the dependent variable was significant, but explained only 3.2 % of the variance in the attitude of the trainee, B(SE) = 2.82(.23), p = .027, showing that high levels of ‘Effectiveness’ in the role model behaviour of GP trainers have a small effect on the attitude of their trainees (Step 3).

There was a discrepancy between trainers and trainees concerning the question whether the trainers discussed the subject of the training course (43.6 vs 15.9 %, p < .01). The trainers and trainees rarely handed out the booklets in daily practice (10.9 and 3.2 %) (Step 2).

## Discussion

A combined CPD/FD course for GP trainers on weight management and their role model function, with the aim of transferring professional competencies as a physician, teacher and role model, resulted in knowledge gains among the GP trainers, even measured using an objective examination and pre-, post-, and delayed post-tests, when compared with a control group. (Step 1) The characteristics that influenced the role model behaviour of the trainers were whether or not they were experienced, the number of patients in the practice and whether the GP trainers practised alone or in partnership. (Step 2) Only a few GP trainers and a very low percentage of the trainees reported using the booklets, indicating restricted use of the information from the training course in daily clinical practice (Step 2), and there was no improvement in trainees’ knowledge or attitude. Finally, a small correlation was found between trainers with better role model behaviour on the items on the ‘Effectiveness’ scale and trainees with the highest scores on attitude. (Step 3)

### Strengths and limitations of the study

The strength of this study lies in the evaluation of the intervention, which was based on an objective measurement of knowledge, completed in the presence of a teacher to ensure objectivity, and we evaluated the role model behaviour of the trainers using the scores of their trainees [12–14] Furthermore we measured the effects of the intervention on the trainees and we evaluated the implementation in daily practice by counting the number of booklets handed out.

There are also limitations to this study. Firstly, there is some doubt about the generalizability of the study. Generalizability was improved by conducting the study in four unrelated training institutes located in different parts of the country to minimize local influences. Although the number of participants in the voluntary intervention group was small, the results from this group showed the same pattern as the results in the mandatory intervention group, thus affirming the value of the properties found.

To reduce the chance of educational interventions at one institute affecting the results of our comparison, we used the GP trainers and trainees at two institutes as controls. With a response of 75 % in the mandatory group and 67 % in the control group, our group of participants seems fairly representative.

Secondly, we found differences in characteristics between the groups. These may have been due to the location of the practices.

Thirdly, a large number of GP trainers failed to follow-up because the attendance of trainers at central curriculum days fluctuated widely; they were often required to work in their practices, shared their presence at training days with co-clinical trainers, or had other obligations.

Finally, only 25 GP trainers chose weight management from a list of different topics and volunteered for the intervention. Previous studies have indicated that GP trainers are not convinced that the treatment of overweight patients is meaningful, which could explain this finding. [25, 43]

### Comparison with existing literature

Several previous CPD/FD studies [15, 17–19] have integrated clinical content with learning about educational methods in voluntary courses for clinical trainers, using different teaching methods; all had positive results, including behavioural changes. Because of the clinicians’ reported lack of time, two courses were, like our training course, short (2–4 h) [15, 18]. The results of these studies were based on self-reporting after the course and three months later. (Step 1) None of these studies used more objective outcome measures or evaluated the course in the target population, (the trainees) or used control groups. As with our findings, one study showed that trainers did not use the written information distributed on the course for their own patients [15]. Another study showed that clinicians routinely overrated their skills and knowledge in self-evaluations, using retrospective pre-programme scores [19]. In our study there was no correlation between the judgment of the trainers regarding whether they were a positive role model and the scores given by their trainees on the RoMAT. There would appear to be a discrepancy between the trainers’ own assessment of their capacities and the observations of the trainees.

Consequently, our limited positive findings may be due to the fact that, instead of relying on the possibly overoptimistic self-evaluation by the trainers [44], we used a more objective means of assessment: objective measurements amongst trainers and trainees, measurements in the training practice, and evaluation of the trainers by the trainees. (Step 2)

Some of the characteristics of the trainers appear to influence role model behaviour. Being more experienced had an influence on the ‘Effectiveness’ scores, implying that being more experienced relates with the ability to meet the learning needs of trainees [3]. Meanwhile, trainers who work alone and were not distracted by colleagues in their practice showed better role model behaviour on the ‘Caring Attitude’ component, thus appearing to be more open toward their trainees [41]. Higher patient numbers in practices (i.e. busier physicians) reduced both scores, leading to less positive role model behaviour. Previous studies found that reserving time for the trainee is a requirement for positive role modeling [3, 42] (Step 2).

The aim of the CPD/FD course, of improving professional competences of the trainee through a cascade effect, was not achieved. Although the first step, that of improving the clinical, teaching and role modelling competences of the trainer, was partly achieved, only a small effect was found for the influence of positive role modelling, and no significant effect on practice behaviour, handing out the booklets. Various reviews [12–14] have discussed quality and composition as factors in the effectiveness of the CPD/FD course. Our study showed that there is a greater loss of competences after the first step, from trainer to training practice and to trainee.

The absence of any knowledge gains on the part of trainees could be explained by the discrepancy between trainers and trainees regarding the question whether the trainers discussed the subject of the training course, implying an overestimation by the trainer and therefore less of a focus on conveying knowledge or, even more importantly, poor receptiveness by the trainee if the topic was not of interest to them at that point in their training. However, the lack of improvement in attitudes toward weight management in our study was in line with previous studies that used the same questionnaire [37, 38] or the same line of questioning [45, 46]. This trend seems to imply that education and the acquisition of more knowledge do not necessarily lead to a better attitude [29]. Finally, although positive role modelling seems to be related to more positive attitudes among trainees, no improvements in role model behaviour could be established and thus no effect on the achievement of the trainee. This may originate from the discrepancy between the trainers’ and the trainees’ evaluation of the trainer as a positive role model. An overestimation of their own positive role modelling may have led to trainers not being motivated to improve their role model behaviour, but also shows the necessity of feedback from trainees on the role modelling of trainers (Step 3).

## Conclusions

Our study analyzed the effects of a CPD/FD course for clinical trainers, by surveying the target population, the trainees. The course integrated knowledge, attitudes, and role modelling, and proved to be a first step toward improving the knowledge of the trainer, but did not result in improved overall professional outcomes for the trainees [47]. Some of the characteristics of the clinical trainer did influence their role model behaviour and there was a small correlation between more positive role model behaviour and positive attitudes among trainees.

### Implications for future courses and research

More research is necessary to establish how to improve the effectiveness of train-the-trainer courses, encouraging clinical trainers to maximize their awareness of conveying knowledge and modelling the correct behavior [3, 4], as well as to determine how to maximize the professional gains for trainees [6] and evaluate such training objectively in the training practice and target population [13, 14].

Essentials

- Integrating knowledge, attitude, and role model behaviour in one course leads to improvements in the knowledge of clinical trainers.
- More research is necessary to establish how to improve the effectiveness of train-the-trainer courses in terms of professional gains for the trainee.
- Effectiveness of courses needs to be assessed by means of objective evaluation among the target population and in daily practice.

## Appendix 1

See Table 5.

**Table 5:** Physicians’ attitudes towards obesity treatment [38, 39]

		Strongly disagree	Disagree	Neutral	Agree	Strongly agree
1	I believe it is necessary to educate obese patients on the health risks of obesity	1	2	3	4	5
2	Obesity is a chronic disease	1	2	3	4	5
3	I make accommodations for obese patients	1	2	3	4	5
4	Obesity is associated with serious medical conditions	1	2	3	4	5
5	Physicians should be role models by maintaining a normal weight	1	2	3	4	5
6	A 10 % reduction in body weight is sufficient to significantly improve obesity-related health complications	1	2	3	4	5
7	I would spend more time working on weight management issues if my time was reimbursed appropriately	1	2	3	4	5
8	I feel competent in prescribing weight loss programmes for obese patients	1	2	3	4	5
9	Most obese patients are well aware of the health risks of obesity	1	2	3	4	5
10	Medications to treat obesity should be limited to short-term (< 3 months) use	1	2	3	4	5
11	Most obese patients could reach a normal weight (for height) if they were motivated to do so	1	2	3	4	5
12	Most obese patients will not lose a significant amount of weight	1	2	3	4	5
13	I have negative reactions towards the appearance of obese patients	1	2	3	4	5
14	If a patient meets the appropriate criteria for obesity surgery, I would recommend an evaluation by a surgeon	1	2	3	4	5
15	Medications to treat obesity should be used chronically	1	2	3	4	5
16	I am usually successful in helping obese patients lose weight	1	2	3	4	5
17	For most obese patients, long-term maintenance of weight loss is impossible	1	2	3	4	5
18	It is acceptable to use ‘scare tactics’ to obtain compliance of the obese patient	1	2	3	4	5
19	I feel uncomfortable when examining an obese patient	1	2	3	4	5
20	It is difficult for me to feel empathy for an obese patient	1	2	3	4	5

## Appendix 2

See Table 6.

**Table 6:** Role Model Apperception Tool (=RoMAT) [40]

S. no.	My clinical trainer	CA/EF^a^	Strongly disagree	Disagree	Neutral	Agree	Strongly agree
1	Has excellent clinical reasoning skills	EF	1	2	3	4	5
2	Conveys empathy for patients	CA	1	2	3	4	5
3	Communicates well with patients and relatives	CA	1	2	3	4	5
4	Understands learners’ needs and is committed to the growths of learners	EF	1	2	3	4	5
5	Establishes rapport with learners	CA	1	2	3	4	5
6	Has a positive attitude towards learners	CA	1	2	3	4	5
7	Demonstrates enthusiasm for one’s work	CA	1	2	3	4	5
8	Is patient	CA	1	2	3	4	5
9	Has a positive interaction with other health care workers	CA	1	2	3	4	5
10	Makes learning exciting and stimulating	EF	1	2	3	4	5
11	Has self-confidence	EF	1	2	3	4	5
12	Is available for learners	CA	1	2	3	4	5
13	Is honest and has integrity	CA	1	2	3	4	5
14	Has leadership qualities	EF	1	2	3	4	5
15	Is aware of his/her role model status	EF	1	2	3	4	5
16	Is nice and easy to work with	CA	1	2	3	4	5
17	Is professionally competent in difficult clinical situations and able to cope with adversity	EF	1	2	3	4	5

^a^Components of the RoMAT: Caring Attitude (CA) and effectiveness (EF)

## References

[1] Byszewski A, Hendelman W, McGuinty C, Moineau G (2012). Wanted: role models—medical students’ perceptions of professionalism. BMC Med Educ.

[2] Janssen AL, Macleod RD, Walker ST (2008). Recognition, reflection, and role models: critical elements in education about care in medicine. Palliat Support Care.

[3] Cruess SR, Cruess RL, Steinert Y (2008). Role modeling—making the most of a powerful teaching strategy. BMJ.

[4] Egnew TR, Wilson J (2011). Role modeling the doctor–patient relationship in the clinical curriculum. Fam Med.

[5] Harden RM, Crosby JR (2000). AMEE Guide No 20: the good teacher is more than a lecturer: the twelve roles of the teacher. Med Teach.

[6] Passi V, Johnson S, Peile E, Wright S, Hafferty F, Johnson N. Doctor role modelling in medical education: BEME Guide No. 27. Med Teach 2013;35(8):e1422–36. 10.3109/0142159X.2013.806982 23826717

[7] Stegeman JH, Schoten EJ, Terpstra OT (2013). Knowing and acting in the clinical workplace: trainees’ perspectives on modeling and feedback. Adv Health Sci Educ.

[8] Bandura A. Social cognitive theory. In: Vasta R. Annals of child development, Vol. 6. Six theories of child development. Greenwich, CT: JAI Press; 1989.

[9] Jochemsen-van der Leeuw HGA, van Dijk N, van Etten-Jamaludin FS, Wieringa-de Waard M (2013). The attributes of the clinical trainer as a role model: a systematic review. Acad Med.

[10] Wright SM, Carrese JA (2002). Excellence in role modeling: insight and perspectives from the pros. CMAJ.

[11] Bowen J, Irby DM (2002). Assessing quality and costs of education in the ambulatory setting: a review of the literature. Acad Med.

[12] Steinert Y, Mann K, Centeno A (2006). A systematic review of faculty development initiatives designed to improve teaching effectiveness in medical education: BEME Guide No. 8. Med Teach.

[13] Sorinola OO, Thistlethwaite J (2013). A systematic review of faculty development activities in family medicine. Med Teach.

[14] Leslie K, Baker L, Egan-Lee E, Esdaile M, Reeves S (2013). Advancing faculty development in medical education: a systematic review. Acad Med.

[15] Nieman LZ (1999). Combining educational process and medical content during preceptor faculty development. Fam Med.

[16] Stratos GA, Katz S, Bergen MR, Hallenbeck J (2006). Faculty development in end-of-life care: evaluation of a national train-the-trainer program. Acad Med.

[17] Karg A, Boendermaker PM, Brand PLP, Cohen-Schotanus J (2013). Integrating continuing medical education and faculty development into a single course: Effect on participants’ behaviour. Med Teach.

[18] Green ML, Gross CP, Kernan WN, Wong JG, Holmboe ES (2003). Integrating teaching skills and clinical content in a faculty development workshop. J Gen Intern Med.

[19] Sullivan AM, Lakoma MD, Billings JE, Peters AS, Block SD (2005). Teaching and learning end-of-life care: evaluation of a faculty development program in palliative care. Acad Med.

[20] Wipf JE, Orlander JD, Anderson JJ (1999). The effect of a teaching skills course on interns’ and students’ evaluations of their resident-teachers. Acad Med.

[21] Post RE, Quattlebaum RG, Benich JJ (2009). Residents-as-teachers curricula: a critical review. Acad Med.

[22] Forestlund L, Bjorndal A, Rashidan A (2009). Continuing education meetings and workshops: effects on professional practice and health care outcomes. Cochrane Database Syst Rev.

[23] Mathus-Vliegen EM (2012). Obesity and the elderly. J Clin Gastroenterol.

[24] Must A, Spadano J, Coakley EH, Field AE, Colditz G, Dietz WH (1999). The disease burden associated with overweight and obesity. JAMA.

[25] Jochemsen-van der Leeuw HGA, van Dijk N, Wieringa-de Waard M. Attitudes towards obesity treatment in GP training practices: a focus group study. Fam Pract 2011;28 (4):422–429. 10.1093/fampra/cmq110 21273284

[26] Van Binsbergen JJ, Langens FNM, Dapper ALM (2010). [Dutch College of General Practitioners’ practice guidelines on obesity]. [In Dutch]. Huisarts Wet.

[27] The Counterweight Project Team, McQuigg M, Brown JE, Broom JL, Laws RA. Engaging patients, clinicians and health funders in weight management: the Counterweight Programme. Fam Pract 2008; 25: i79–86. 10.1093/fampra/cmn081 19042914

[28] Fransen GA, Hiddink GJ, Koelen MA (2008). The development of a minimal intervention strategy to address overweight and obesity in adult primary care patients in the Netherlands. Fam Pract.

[29] Dacey M, Arnstein I (2013). The impact of lifestyle medicine continuing education on provider knowledge, attitudes and counselling behaviors. Med Teach.

[30] Hashy RB, Munna RK, Vogel RL, Bason JJ (2003). Does physician weight affect perception of health advice?. Prev Med.

[31] Bocquier A, Verger P, Basdevant A (2005). Overweight and obesity: knowledge, attitudes, and practices of general practitioners in France. Obes Res.

[32] Forman-Hoffman V, Little A, Wahls T (2006). Barriers to obesity management: a pilot study of primary care clinicians. BMC Fam Pract.

[33] Van Berkel H, Bax A (2002). Testing in higher education.

[34] Van Avendonk MJ, van Binsbergen JJ, Langens FN, Boukes FS, Goudswaard AN (2010). Summary of the Dutch College of General Practitioners’ practice guidelines on obesity. [Article in Dutch, Abstract in English]. Ned Tijdschr Geneeskd.

[35] Partnerschap Overgewicht Nederland. [Guidelines on care for obesity]. [In Dutch] 2011. http://www.partnerschapovergewicht.nl/site_files/uploads/PON_Zorgstandaard_Obesitas_2011_A4_v1%2004.pdf. Accessed 6 Mar 2014.

[36] [CBO Guidelines on diagnostics and treatment of obesity in adults and children]. [In Dutch] 2008. http://www.diliguide.nl/document/530/obesitas-diagnostiek-en-behandeling-van-obesitas.html. Accessed 6 Mar 2014.

[37] Foster GD, Wadden TA, Makris AP (2003). Primary care physicians’ attitudes about obesity and its treatment. Obes Res.

[38] Davis NJ, Shishodia H, Taqui B, Dumfeh C, Wylie-Rosett J (2007). Resident physician attitudes and competence about obesity treatment: need for improved education. Med Educ.

[39] Jochemsen-van der Leeuw HGA, van Dijk N, Wieringa-de Waard M (2014). Assessment of the clinical trainer as a role model; a role model apperception tool (RoMAT). Acad Med.

[40] Hojat M, Xu G (2004). A visitor’s guide to effect sizes. Adv Health Sci Educ.

[41] Wright S (1996). Examining what residents look for in their role models. Acad Med.

[42] Wright SM, Kern DE, Kolodner K, Howard DM, Brancatie FL (1998). Attributes of excellent attending-physician role models. N Engl J Med.

[43] Ruelaz AR, Diefenbach P, Simon B, Lanto A, Arterburn D, Shekelle PG (2007). Perceived barriers to weight management in primary care—perspectives of patients and providers. J Gen Intern Med.

[44] Rubak S, Mortensen L, Ringsted C, Malling B (2008). A controlled trial of the short- and long-term effects of a train the trainer course. Med Educ.

[45] Ip EH, Marshall S, Vitolins M (2013). Measuring medical student attitudes and beliefs regarding patients who are obese. Acad Med.

[46] Block JP, DeSalvo KB, Fisher WP (2003). Are physicians equipped to address the obesity epidemic? Knowledge and attitudes of internal medicine residents. Prev Med.

[47] Reilly B (2007). Inconvenient truths about effective clinical teaching. Lancet.

[48] The Ethical Review Board of the NVMO (Dutch Association for Medical Education). http://www.nvmo.nl/ethische_toetsing_onderzoek. Accessed 6 Mar 2014.

